# Retraction: Bhosale, S.V., et al. pH Dependent Molecular Self-Assembly of Octaphosphonate Porphyrin of Nanoscale Dimensions: Nanosphere and Nanorod Aggregates. *Int. J. Mol. Sci.* 2011, *12*, 1464–1473

**DOI:** 10.3390/ijms22031327

**Published:** 2021-01-29

**Authors:** International Journal of Molecular Sciences Editorial Office

**Affiliations:** MDPI, St. Alban-Anlage 66, 4052 Basel, Switzerland; ijms@mdpi.com

The journal retracts the article, “pH Dependent Molecular Self-Assembly of Octaphosphonate Porphyrin of Nanoscale Dimensions: Nanosphere and Nanorod Aggregates” [1], cited above. 

Following publication, concerns were brought to the attention of the publisher regarding the figures. Compared with the raw data, Figure 5a appears to have been manipulated. This has brought about uncertainty regarding the scientific conclusions. In addition, this paper [1] was submitted without the permission of all the co-authors. Therefore, this paper [1] is retracted and shall be marked accordingly. The Editor-in-Chief of the *International Journal of Molecular Sciences* has approved this retraction. 

We apologize to our readership that this went undetected until now.
